# Neurolinguistic Relativity: How Language Flexes Human Perception and Cognition

**DOI:** 10.1111/lang.12186

**Published:** 2016-06-19

**Authors:** Guillaume Thierry

**Affiliations:** ^1^ School of Psychology and Centre for Research on BilingualismBangor University

**Keywords:** language, perception, cognition, Whorfianism, event‐related brain potentials

## Abstract

The time has come, perhaps, to go beyond merely acknowledging that language is a core manifestation of the workings of the human mind and that it relates interactively to all aspects of thinking. The issue, thus, is not to decide whether language and human thought may be ineluctably linked (they just are), but rather to determine what the characteristics of this relationship may be and to understand how language influences—and may be influenced by—nonverbal information processing. In an attempt to demystify linguistic relativity, I review neurolinguistic studies from our research group showing a link between linguistic distinctions and perceptual or conceptual processing. On the basis of empirical evidence showing effects of terminology on perception, language‐idiosyncratic relationships in semantic memory, grammatical skewing of event conceptualization, and unconscious modulation of executive functioning by verbal input, I advocate a neurofunctional approach through which we can systematically explore how languages shape human thought.

## Introduction

Think of a frog. Even better, perhaps, don't. Here, language—in this case, a noun supported by a verb—will have prompted a thought in your mind. Does language have a strong relationship to thought? The second instruction above (don't think of a frog) is perhaps an even better illustration of the link: Not only does language appear inherently and implacably connected to thought, but the connection seems to escape our control entirely. It is impossible for one not to think of something called up by language. Thus, language triggers thought, and it does so whether we like it or not, provided we are familiar with the code. Language is undoubtedly one of the most exquisitely sophisticated and powerful products of the human mind. Its importance in the development of human civilization, society, and science hardly needs introducing or highlighting. Even though the language‐thought binding contingency appears intuitive to many, some scholars and thinkers have dismissed the premises of linguistic relativity—an equally intuitive correlate of the contingency—as linguistic idealism (see Jackson, 1991).

Casasanto (2008) reminds us that language is naturally not the same as thought, because thought preexists language, both ontologically and phylogenetically. Animals manifest forms of cognitive processing (e.g., perception, attention, memory, problem solving, planning, adaptive execution) that resemble thought processes seen in humans (e.g., Gallistel, 1989). Preverbal infants anticipate, direct their attention, draw inferences, and thus manifest thought outside language (Feigenson, Dehaene, & Spelke, 2004; Gordon, 2004). But after having agreed that we must leave the idea of a language‐thought equivalence behind, we are not yet out of the woods. We can still come across the “big bad Whorf” (Casasanto, 2008). One thing is to recognize that thought exists outside language and thus that it likely coexists with language. Another is to wander at the other end of a conceptual continuum and consider that thought might be entirely independent of language. Does Pinker (2007) use a strawman strategy to counter and ridicule the linguistic relativity hypothesis? If language does not equate thought, must we readily adopt the extreme opposite view and consider language‐thought independence as a de facto alternative? That is wrong, though, all wrong. Thinking that language may be entirely disconnected from thought is an example of what deserves to be called *reductio ad absurdum*.

People who speak different languages appear to conceive of the world somewhat differently and diversity of thought, perspective, and innovation seem intuitively linked to naturally occurring language diversity. Why is it, then, that many scholars in science, linguistics, and philosophy (perhaps in these fields more than elsewhere) are skeptical, if not overtly critical, of the idea that languages may go hand in hand with different ways of not only conceiving and paying attention to the world, but also literally holding different perceptions? Perhaps the most basic argument against a strong language‐thought binding contingency concerns the commonly advocated existence of universal concepts. If such concepts exist, then thought must be separable from language. But then, reciprocally, if language is narrowly married to thought, and if together they are somewhat idiosyncratic to each and every human mind, how do we know that universal concepts are indeed universal? For instance, Jackson (1991) asks, “Can the French not tell the difference between sheep and mutton, because they only have one word for them?” (p. 209). Indeed, even though native speakers of French use the label *mouton* for both the meat derived from the sheep and the animal itself, they can conceptualize this distinction. If the meat–animal distinction is shared by native speakers of both French and English, then language arguably fails to shift the concept. However, because French and English differ in the way they refer to the meat and the animal it derives from, one can reasonably hypothesize that this distinction is, in fact, conceptually different for the speakers of the two languages (e.g., a sheep may be considered more primarily as a source of food by French speakers than English speakers). We thus find ourselves prisoners of a circular reasoning loop: Language and thought seem bound up with one another, but then there appear to be shared concepts that transcend language diversity. We cannot, however, be sure that these concepts are shared if we cannot conceptualize something independently of a particular language or languages, and so on.

Such circular reasoning has profoundly hindered progress in characterizing the nature of the language‐thought binding contingency and partly explains the stark skepticism that has stigmatized linguistic relativity. Breaking out of the loop thus requires a demonstration that language and thought are intertwined even when one ventures into territories of human cognition that are as far from language as we can conceive. One such territory is perception. Indeed, empirical research testing the linguistic relativity hypothesis has progressively drifted toward the testing of nonverbal perception, in the hopes of identifying processing differences that do not trivially derive or relate to (consciously accessible) language distinctions, but that can rationally and indirectly be linked to the same. However, such empirical studies (e.g., Boroditsky, 2000, 2001; Gentner, Imai, & Boroditsky, 2002; Lupyan, 2008; Lupyan & Spivey, 2010; Meteyard, Bahrami, & Vigliocco, 2007) have failed to convince the skeptics (e.g., Gleitman & Papafragou, 2005; Klemfuss, Prinzmetal, & Ivry, 2012; Pinker, 2007), perhaps because (a) they hardly ever avoid language reference entirely, (b) they fail to test perception, or (c) because of a combination of (a) and (b).

Studies relying on behavioral measures (such as reaction times and response patterns) are notoriously susceptible to verbal interference and late, postperceptual strategic effects, which may explain why reports of such effects are often followed by publications of counterevidence or null effects studying the same contrasts (e.g., Firestone & Scholl, 2014; January & Kako, 2007). Humans can (and do) silently verbalize all the time and thus responses from conscious decisions in supposedly nonverbal perceptual tasks can never be deemed free of verbal contamination. Rather than having differently structured minds, then, speakers of different languages may have their behavior nudged by grammar or terminology in ways that reflect properties of their native language (Pinker, 1997). This pertinent criticism applies to most empirical studies that attempt to draw conclusions from behavioral measures. How, then, can potential effects of language structure on perception be tested? This requires an investigation method that can tackle stages of mental processing that are sufficiently early, unconscious, and automatic, so as to not be directly affected by online aspects of language processing. There is thus no alternative but to test the hypothesis using physiological correlates of perception that derive from brain activity.

A fully interactive, nonselective account of human brain physiology is largely inconsistent with modular views positing a given faculty (such as language) as encapsulated and relatively independent vis‐à‐vis other specialized brain systems such as perception, object categorization, or motor control (Barsalou, 2008). As highlighted by Pulvermüller (1999) and in line with the theoretical tenets of connectionism and Hebbian theory (Hebb, 1949), the human brain is a richly connected network of neurons, in which functional subnetworks recruit distributed cells firing in coincidence (cell assemblies) rather than anatomically and/or functionally distinct modules. If one considers that connectivity within the brain—involving forward connections and feedback loops—spans the entire cortex and internal ganglia, it soon becomes evident that making a distinction between language and the rest of the mind is essentially meaningless. Making such a distinction implies that language and mind are two ensembles that can be delimited, as if one could draw a line between the two, or trace a line around language within the mind. This is misleading both from an anatomical and a neurophysiological viewpoint. First, it is well established that high‐level executive functions such as planning and attention modulate perceptual processing at the most basic levels of processing (McAdams & Reid, 2005). Second, to this day, there is little or no evidence for language‐specific regions in the human brain (Démonet, Thierry, & Cardebat, 2005; Price, Thierry, & Griffiths, 2005). Indeed, areas of the cerebral cortex, inner ganglia, and cerebellum involved in language processing are also activated by various nonverbal auditory and visual stimuli presented in a variety of experimental contexts (e.g., Thierry, Giraud, & Price, 2003; Thierry & Price, 2006). Even though some recent studies have offered evidence in favor of what may be construed as microselectivity in areas often associated with language processing (Fedorenko, Duncan, & Kanwisher, 2012), the spatial and temporal resolution of functional neuroimaging remains largely insufficient to establish any selectivity at the macrostructural level. Third, there is no shortage of empirical evidence showing interaction between language and other perceptual‐cognitive processes, such as emotion (Wu & Thierry, 2012), decision making (Costa, Foucart, Arnon, Aparici, & Apesteguia, 2014; Gao, Zika, Rogers, & Thierry, 2015; Keysar, Hayakawa, & An, 2012), or inhibitory control (Wu & Thierry, 2013).

In sum, neurophysiology and cognitive neuroscience suggest that language and thought are intrinsically bound together, and thus that language likely influences thought. An example of the mechanism through which this can occur is the label‐feedback hypothesis put forward by Lupyan (2012). Lupyan proposed that a category name becomes associated with distinctive features of the category it denotes as soon as it is learned. The activation of this name or label then results in the modulation of lower‐level perceptual processes via feedback looping, filtering, and improving the bottom‐up flow of information through the perceptual system. Naming an object would thus lead to more categorical and less idiosyncratic representations and effectively lead to the perceptual grouping of objects denoted by the same category label. This model, however, makes the strong assumption that language representations (and the neural network underpinning them) modulate low‐level perceptual processing in real time rather than by means of more durable structural and functional reorganizations. Furthermore, the label‐feedback hypothesis accounts for lexical effects, but says little or nothing about the impact on perception of other structural properties of language such as grammar and syntax. If the language–thought binding contingency seems obvious and pervasive from a cognitive neuroscience perspective, why spend time demonstrating its existence? I would argue that relativist research has two main objectives: (a) characterizing the nature of the effects and mechanisms through which language influences other aspects of cognition and (b) establishing whether there exist conditions in which thought may actually be independent of language.

As long as the phenomena studied empirically in linguistics, psycholinguistics, neurolinguistics, and cognitive science require, or merely encourage, conscious processing and verbalization, it will be impossible to establish an effect of language on nonverbal thinking. Progress thus requires evidence that minimizes the chances of a contamination by explicit top‐down strategies, which are likely to prompt language processing (overtly or implicitly) during tasks that are supposedly nonverbal. In this sense, most behavioral studies provide insufficient evidence, because the effects reported can always be construed as a mere effect of language on language (Pinker, 2007; Slobin, 1996). The need for a neurophysiological approach becomes more and more clear: Observing modulations of neural activity relating to perceptual or unconscious evaluation of nonverbal stimuli that can be predicted on the basis of definitional, contrasting properties of languages must be the best evidence that language shapes human thought.

I am hopeful that this outlook challenges the idea that there are instances in which language stands alone, disconnected from perception, attention, memory, executive control, or thought, whatever definition one chooses to lend them. Here, I discuss findings from three domains of research, which I believe strongly undermine the idea that language may stand apart from the rest of our cognitive make‐up: (a) color and object categorization, (b) motion conceptualization, and (c) executive function.

## Color and Object Categorization

The firm determination to submit to experiment is not enough; there are still dangerous hypotheses; first, and above all, those which are tacit and unconscious. Since we make them without knowing it, we are powerless to abandon them. (Henri Poincaré, 1913, p. 134)

Notwithstanding the disputable nature of conclusions from behavioral studies relying solely on overt decision data, a host of recent studies have convincingly demonstrated that lexical and grammatical information affects domain‐general cognitive processes. For example, color terminology has been shown to influence categorical perception of color in monolingual and bilingual speakers (Franklin et al., 2008; Gilbert, Regier, Kay, & Ivry, 2006). Athanasopoulos and colleagues, in particular, have provided empirical evidence that native speakers of Greek, who in their language have two basic color terms for light and dark blue (*ghalazio* and *ble*), perceive these two shades of blue as more distinct than do native speakers of English, whose language has only the basic color term *blue* (Athanasopoulos, 2009; Athanasopoulos, Damjanovic, Krajciova, & Sasaki, 2011). Such effects have been found both in tasks inviting participants to identify prototypical colors corresponding to a particular term and similarity judgment tasks (Athanasopoulos et al., 2011).

To address the core question of possible online access to language representations during overt behavioral tasks requiring conscious evaluation of color features, we chose to record event‐related brain potentials in participants, who were monitoring colored shapes in a very simple shape detection task. Event‐related potentials (ERPs) are derived from electroencephalographic (EEG) activity recorded continuously throughout an experimental session from the surface of the scalp of a participant, who is usually presented with a series of visual or auditory stimuli. Real‐time continuous EEG is essentially impossible to interpret as is, at least when it comes to making inferences about the cognitive processes involved. In order to purify the activity specific to a given stimulus category or cognitive operation, the continuous EEG recording is split into epochs, using stimulus onset time as a temporal reference. By averaging EEG signals collected over several trials (generally at least 30), brain activity that is unrelated to the particular stimulus presented or the particular cognitive operation performed by the participant is blurred and tends to fade into background activity, whereas those variations of electrical potentials that specifically relate to the stimuli presented tend to be repeated from one trial to the next and thus to be revealed through averaging. ERPs offer a unique opportunity to study the average modulation of activity produced by the brain in response to a class of stimuli and/or a particular cognitive task. The temporal resolution of the method is very high (in the order of the millisecond), but it provides little or no information regarding the source of the signal in the brain.

We presented native speakers of Greek and English with streams of simple shapes (mostly circles and, infrequently, squares) filled in light blue, dark blue, light green, or dark green and instructed them to press a designated button when they saw a square. Participants’ attention was thus drawn to shape rather than color. Such an experimental procedure, the so‐called oddball paradigm, has been used extensively both in the auditory and the visual modalities to study an automatic and unconscious response of the brain to stimulus perceptual deviance (a detectable difference between frequent and rare stimuli)—mismatch negativity (Czigler, 2014; Näätänen, Kujala, & Winkler, 2011). We predicted that the existence of two basic color terms in Greek for light blue and dark blue would result in a visual mismatch negativity (vMMN) of larger magnitude for the blue contrast than the green contrast. The light green/dark green contrast was used as a control condition, because Greek (like English) has only one basic color term for green (*prasino*). Therefore, we expected the contrast between light green and dark green to elicit vMMNs of similar magnitude in the two groups. And, indeed, we found the predicted three‐way interaction between participant group, color hue, and stimulus deviancy (Thierry, Athanasopoulos, Wiggett, Dering, & Kuipers, 2009). We concluded that a lexical distinction in a given language leads to greater perceptual discrimination in individuals who possess this distinction, as compared to speakers of a language that lacks the distinction. We argued that this effect is not linked to online activation of verbal representations in the mind of the participant (see below), but rather constitutes evidence for a perceptual distinction established through repeated exposure to color terms that constantly highlight color contrasts.

Some of our reviewers and a number of colleagues raised questions at the time about this finding, most notably: (a) How do we know that the process is unconscious? (b) Why could this not be explained by online lexical access, the result being another example of online effects of language that do not imply the long‐term shaping of nonverbal representations by language? and (c) How do we know that the effects found are not merely due to the different environments in which the individuals of the two compared groups were raised? In other words, how do we know that this is not merely a question of environmental experience rather than a genuine effect of terminology and language use? Here are tentative answers to these questions:

- We debriefed our participants very carefully at the end of each experiment, and we were surprised to discover that most failed to recall that standard stimuli (circles) could have different colors within any given block. Most did recall (correctly) that squares could change color (i.e., be light blue or dark blue within a block), but they failed to remember that the circles, too, could change color within a block, even though this was the perceptual change at the origin of the vMMN. The fact that participants could not recall the critical manipulation at the origin of the vMMN modulation suggests that they were not paying much attention to the critical stimuli and that the processing of color deviancy was mostly automatic.
- Studies of online speech production using ERPs have shown that participants in optimal conditions (after training) show the earliest effects of lexical frequency at around 200 milliseconds after stimulus onset in picture naming (Costa, Strijkers, Martin, & Thierry, 2009; Strijkers, Costa, & Thierry, 2010). In addition, when participants are not required to name pictures, but rather make semantic decisions about them, lexical frequency effects are observed much later, presumably because lexical properties of picture names are not critical to perform a semantic decision task (Strijkers, Holcomb, & Costa, 2012). Taken together, these studies suggest that specific lexical information is only available after 200 milliseconds following the presentation of a picture, even when picture names have been practiced and naming is directly required. The vMMN effect recorded in Thierry et al. (2009) started before 200 milliseconds and occurred in circumstances when the critical stimuli required no naming, let alone full perceptual evaluation, which suggests that it was not under online influence of language representations. Furthermore, when we investigated the latency and amplitude of the P1 (an early peak of ERPs classically associated with early visual processing), we again found a striking dissociation between groups: British participants showed a close overlap of amplitude and latencies between P1s elicited by stimuli of matched lightness (light blue and green on the one hand, and dark blue and green on the other), but Greek participants showed a marked P1 dissociation between the four stimuli as well as substantially increased variance. These differences are very unlikely to be reducible to online effect of language processing.
- Greek participants were likely to have experienced different shades of blue in their lives compared to locally sourced native speakers of English, given the marked meteorological contrasts between Greece and Wales. However, and even though this is not a definitive argument, the environment in Wales and the United Kingdom generally boasts an extraordinary palette of green shades, whereas the same can hardly be said of blue which is, after all, rather infrequent in the United Kingdom. Therefore, if the larger vMMN contrast found for blue than green in the native Greek participants could be accounted for by the naturally occurring variety of blue shades in the environment in Greece, one could reasonably predict a larger vMMN contrast for green than blue in the British participant group. However, this was not observed in our data.

Shortly after the publication of this first study, we realized that our Greek group of 20 participants could be split into two groups of 10 with markedly different lengths of stay in the United Kingdom. We thus set out to test whether a longer stay in the United Kingdom, naturally correlated with increased proficiency in English, would lead to a reduction in the vMMN effect, because the use of the word blue to refer to both *ghalazio* and *ble* might have led to a dimming of the perceptual contrast between the two colors. We compared three groups: 10 native speakers of Greek who had spent an average of 7.2 months (5–12) in the United Kingdom (short stay), 10 native speakers of Greek who had spent an average of 42.6 months (18–60) in the United Kingdom (long stay), and a group of 10 native speakers of English randomly selected from the 20 tested in the 2009 study. Recalculated vMMN results in the new participant groupings revealed that dark and light blues were not only perceived as more similar by long‐stay Greek participants, but the vMMN effect elicited by the blue contrast was also markedly reduced, as compared to that elicited in short‐stay bilinguals (Athanasopoulos, Dering, Wiggett, Kuipers, & Thierry, 2010). These results strongly suggest that terminology not only drives categorical distinction at a perceptual level, but also that this process is eminently plastic in nature, modulated by language exposure and usage.

Following these studies in the domain of color perception, we wondered if a similar phenomenon could be observed in the domain of object categorization. In collaboration with Bastien Boutonnet, we designed an experiment similar to the color study contrasting pictures of cups and mugs, one picture being the frequently presented standard and the other being the infrequent deviant (Boutonnet, Dering, Viñas‐Guasch, & Thierry, 2013). Infrequent pictures depicting a bowl were the targets, which participants had to detect by pressing a button. We tested 14 native speakers of English, who had no difficulty naming the objects as *cup* and *mug* and 13 native speakers of Spanish in Spain, who all called both objects *taza*. We predicted a two‐way interaction between group and deviancy, such that the vMMN difference between cups and mugs would be greater in the English natives than the Spanish natives. Indeed, we found essentially no vMMN amplitude difference between cups and mugs in the Spanish group and a significant difference in native speakers of English. It must be kept in mind that ERP markers of perceptual differences between objects differentiated the two categories reliably in both groups of participants, that is, the P1 peak of ERPs elicited by cups and mugs, averaged irrespective of frequency status in the oddball experiment, elicited a P1 difference that was very similar in the two groups, showing that the two objects were perceived as different, and to a similar extent by speakers of each language. Indeed, it would be ludicrous to think that because two objects are named using the same word, they could be taken to be visually indistinguishable. However, during early stages of visual categorization, these objects are less distinguishable among individuals who use the same term to designate them than those who usually use distinct terms, and in that sense, it remains fundamentally a question of perception.

Effects such as those reviewed above are eminently predictable for one who is positively predisposed toward the premises of the linguistic relativity hypothesis: A difference in terminology relates to a difference in perceptual processing. A stronger argument in favor of linguistic relativity, then, would be to identify not merely concepts that can be distinguished on the basis of specific lexical contrasts, but rather concepts that are related in a nontrivial fashion, for instance, due to the existence of links that exist at a predominantly linguistic level and have relatively low incidence at a conceptual level. The concepts of *horse* and *sea*, for instance, can be considered only mildly related from a conceptual viewpoint and probably not more so than *bike* and *sea*. *Horse* and *sea*, however, are formally related in English through the existence of the compound word *seahorse*. In the study by Boutonnet, McClain, and Thierry (2014), we tested whether such relations between words idiosyncratic to a language would yield predictable links between corresponding nonverbal representations and concepts. We presented participants undergoing ERP recordings with picture pairs that were either related in meaning, unrelated in meaning, or arbitrarily related because of the existence of a compound word comprising the names of the objects depicted by the two pictures. We quantified picture‐to‐picture priming using the N400, a wave of ERP known to be modulated in amplitude by semantic priming (Kutas & Federmeier, 2011). As expected, we found that related pictures elicited an N400 of reduced mean amplitude as compared to unrelated pictures. Surprisingly, however, while pictures derived from words forming a compound and presented in the same order (as in *sea*–*horse*) elicited an N400 response similar to unrelated pictures, the N400 was significantly reduced when the pictures were presented in the reverse order (as in *horse*–*sea*). We interpreted this result as evidence that the two concepts (e.g., *horse* and *sea*), presented nonverbally and visually, are abnormally related in semantic memory, because they primed one another to a greater extent than otherwise unrelated concepts (e.g., *horse* and *smoke*), probably due to the existence of a compound word crystallizing a formal link within the lexicon (e.g., *seahorse*). It may be surprising that we did not find such priming effect when the pictures were presented in the same order as words within the corresponding compound (e.g., *sea*–*horse*). We interpreted these findings as a clash between conceptual priming and access to the meaning of the actual compound (e.g., *seahorse*), which was unrelated and thus conflicted with the concepts activated by the pictures. This study broke new ground in neurolinguistic approaches to linguistic relativity by revealing semantic associations between concepts driven mainly by lexical relations idiosyncratic to a particular language and thus nontrivially reliant on the *signifiant*–*signifié* relationship of Saussure (Saussure, Bally, Sechehaye, & Riedlinger, 1916).

## Motion Conceptualization

Users of markedly different grammars […] are not equivalent as observers but must arrive at somewhat different views of the world. (Benjamin Lee Whorf, 1956, p. 221)

One essential question, much closer to Whorf's original speculations, is whether effects of language on perception through a mind‐shaping effect of grammar could be more entrenched than those originating in terminology. Beyond linguistic observations suggesting that grammatical number and grammatical gender may alter object categorization in observers of different languages (Athanasopoulos & Kasai, 2008; Boutonnet, Athanasopoulos, & Thierry, 2012; Cubelli, Paolieri, Lotto, & Job, 2011; Saalbach, Imai, & Schalk, 2012), a paradigm that has attracted a lot of attention is that of motion perception, because of the rich diversity of the way in which different languages deal with, for example, path, goal, direction, and manner of motion.

Some languages (e.g., English) tend to encode manner of motion within the verb (compare *to walk* and *to stroll*, or *to run* and *to sprint*) whereas others (e.g., French; Talmy, 1985) will more often optionally encode manner through the addition of adverbs (compare *marcher* and *marcher lentement*, or *courrir and courrir très vite*, which are tentative French translations of the previously exemplified English verbs). Apart from the fact that individuals who speak a manner‐oriented language will (rather expectedly) be more inclined to linguistically encode manner when describing motion events (Papafragou, Hulbert, & Trueswell, 2008), the features of the language have been suggested to mildly influence behavior in eye‐tracking experiments (Flecken, Carroll, Weimar, & von Stutterheim, 2015; Papafragou et al., 2008) and in a variety of categorization, matching, and recognition tasks (Gennari, Sloman, Malt, & Fitch, 2002; Kersten et al., 2010; Papafragou, Massey, & Gleitman, 2002).

Another key example of the way in which grammatical idiosyncrasies of different languages affect linguistic encoding strategies and, correlatively, conceptualization of motion events, pertains to the domain of grammatical aspect. Studies by von Stutterheim and Nüse (2013), von Stutterheim, Andermann, Carroll, Flecken, and Schmiedtova (2012), and Flecken, von Stutterheim, and Carroll (2014), for instance, have shown a relationship between grammatical aspect and attention to endpoints when individuals observe motion. Indeed, languages can be distinguished on the basis of their systematic or optional encoding of aspect within verbs (Slobin, 2006). When asked to describe an incomplete motion event (e.g., a video clip showing a woman walking on a road toward a car but not reaching the vehicle), speakers of nonaspect languages (e.g., German or Swedish) tend to specify the action's goal, or the endpoint, as compared to speakers of aspect languages (e.g., English or French), who tend to keep to the description of the motion itself and are thus less likely to mention the endpoint (Bylund & Athanasopoulos, 2014; Bylund, Athanasopoulos, & Oostendorp, 2013).

Perhaps the most radical demonstration of the impact of language aspect on conceptualization is that recently offered by Athanasopoulos et al. (2015) who showed how fluent German‐English bilinguals can switch back and forth between the goal‐orientation preference of native speakers of either of their two languages, depending on language context. Strikingly, when bilingual German‐English participants are asked to perform a verbal interference task (counting backwards) in either English or German while categorizing video clips depicting motion events, they display the goal‐orientation preference of the language not in use, as if that language was available to shape selection behavior!

While substantial behavioral evidence is now available to show that motion event conceptualization depends on the properties of the native language and the immediate language context, it remains the case that observing motion events depicted by video clips or monitoring of eye movements using eye‐tracking during stimulus observation cannot exclude covert verbalization, and thus the possibility of online influences of language, which again raises the spectre of the effect of language on language criticism. Here, too, more compelling evidence in support of the strong relativist viewpoint (Gentner & Goldin‐Meadow, 2003; Gumperz & Levinson, 1996; Lucy, 1997) must come from cognitive neurolinguistics, using experimental procedures that are less susceptible to covert language involvement and that rely on direct neurophysiological measures of cognitive processing more readily linked to conceptualization.

In a recent study by Flecken, Athanasopoulos, Kuipers, and Thierry (2015), this was precisely what we aimed to do. We presented two groups of native speakers of German or English with video animations of a black dot travelling along a trajectory (straight or curved) toward a shape (square or hexagon). Given that speakers of English, an aspect‐language, are drawn to pay attention equally to trajectory and endpoint, whereas speakers of German, a nonaspect language, tend to pay more attention to endpoints, we expected the perceptual saliency of the endpoint to be greater in native speakers of German than native speakers of English. We engaged our participants in a motion event‐picture matching task in which animations were used as primes and were followed by picture targets symbolizing the motion events. In 75% of trials, the animation prime (e.g., dot moving along a straight line toward a square) was followed by a target picture featuring both a mismatched trajectory (e.g., a curve) and a mismatched endpoint shape (e.g., a hexagon; mismatch condition); in 10% of trials, the trajectory depicted in the picture target matched that of the dot in the animation prime (trajectory match); in 10% of trials, the shape in the picture target matched the endpoint shape of the animation prime (endpoint shape match); and in only 5% of trials, both the trajectory and the endpoint shape matched that of the animation prime (full match). Participants were instructed to press a button only in the full match condition. This design conformed to that of an oddball paradigm geared toward probing conceptualization and conscious monitoring because the relevant animation characteristics (trajectory and endpoint) were directly relevant for task completion. We thus expected to see a P3b wave of event‐related brain potentials in the full‐match condition, because this wave is well known to index conscious detection of infrequent target stimuli among frequent stimuli. Critically, the amplitude of the P3 elicited in partial match conditions served as an index of the perceived importance of trajectory and endpoint information, respectively. Endpoint‐match stimuli elicited greater P3 amplitude than trajectory‐match stimuli in native speakers of German tested in Germany, but no differences between the P3s elicited by endpoint‐match and trajectory‐match stimuli were found in native speakers of English tested in the United Kingdom. Furthermore, a behavioral testing procedure conducted in similar language groups failed to show any performance difference between groups. This study is probably the first to show that grammatical properties of the native language affect motion event conceptualization in a systematic fashion, even when language involvement is unlikely, given the nature of the stimuli and task, and when categorization behavior is not overtly biased by the instructions.

## Executive Function

But if thought corrupts language, language can also corrupt thought. (George Orwell, 1946, p. 167)

Beyond effects of language on perception and categorization, a critical question is whether language can influence or even constrain aspects of executive function and action selection. Language experience is known to affect cognitive abilities. For example, bilinguals have been shown to outperform monolinguals in a number of nonlinguistic tasks (e.g., the Simon task and the Attention Network Test) that measure various aspects of executive function (Bialystok, Craik, Klein, & Viswanathan, 2004; Costa, Hernández, & Sebastián‐Gallés, 2008). One explanation for this apparent advantage is that managing two languages requires constant selection of words in the intended language and inhibition of words from the unintended language, processes that are thought to engender a highly efficient control mechanism (Bialystok & Feng, 2009; Green & Abutalebi, 2013). However, groups of bilinguals and monolinguals differ not only in language ability but also in other respects, such as socioeconomic status or ethnic origin and, indeed, these factors have been suggested to account for the bilingual advantage (Costa, Hernández, Costa‐Faidella, & Sebastián‐Gallés, 2009; Morton & Harper, 2007; Sabbagh, Xu, Carlson, Moses, & Lee, 2006). Unlike monolinguals, bilinguals can either use only a single language (monolingual context) or both their languages (bilingual context) in any given interaction. If bilingualism bestows a generic and sustained executive control advantage to bilinguals, enhanced cognitive control should be observed independently of language context. If, on the contrary, the advantage is context dependent, enhancement should be greater in a bilingual than in a monolingual context.

Wu and Thierry (2013) tested whether language context modulates executive functioning in Welsh‐English bilinguals by measuring interference in an adapted version of the flanker task (Fan, McCandliss, Sommer, Raz, & Posner, 2002). Participants were instructed to report the direction of a central arrow (i.e., the target) surrounded by congruent or incongruent flanker arrows. Occasionally, instead of arrows, participants were presented with a word, which they were instructed to ignore. The experiment contained three blocks in which the contextual words to be ignored were in Welsh, in English, or in either of the two languages. Whereas the cognitive interference caused by incongruent trials was of similar magnitude when intervening words were all in one language or the other within a block, the amount of interference decreased (and thus participants were better able to manage incongruent trials) when contextual words were randomly presented in English and Welsh. This study was the first to demonstrate a direct influence of language context on executive function within the same bilingual individuals, which means that inhibitory control can be modulated by simply presenting participants with a mix of words from their two languages, even when word stimuli are irrelevant and participants are instructed to ignore them.

In the domain of decision making, language has been found to influence cognitive and emotional processes that mediate departures from normatively rational choice (Keysar et al., 2012). This is due in part to the fact that language can modify one's emotional state, which in turn can affect various aspects of decision making (Damasio, 1996; De Martino, Kumaran, Seymour, & Dolan, 2006). Damasio (1996) provided compelling clinical evidence that emotion is an integral component of decision making that can lead to suboptimal decisions or even indecision. On the other hand, mental representations are known to be sensitive to established language‐emotion interactions. Wu and Thierry (2012), for instance, reported inhibition of access to native lexical representations by emotionally negative words presented in the second language of Chinese‐English bilinguals: When presented with pairs of English words that concealed a Chinese character repetition in their Chinese translation, a well‐established effect of unconscious access to native translation equivalent representation (Thierry & Wu, 2007; Wu & Thierry, 2010) was not found if the prime word had a negative affective valence, but the anticipated priming was found when primes were affectively positive or neutral. This suggests that emotional aspects of people's decision making, sometimes associated with suboptimal choices, may be susceptible to the language context in which they operate.

Keysar and colleagues (2012), who investigated language‐cognition interactions in decision making under risk, showed how using a foreign language modulates framing and loss aversion when participants choose between risky and safe prospects. In their native language, participants displayed standard risk aversion for dilemmas emphasizing gains, on the one hand, and risk‐seeking behavior for dilemmas emphasizing losses, on the other, but they did not do so in their second language. This and other findings (e.g., Costa et al., 2014) show that making decisions in a second language moderates peoples’ risk‐attitudes by underweighting larger gains and losses, which is consistent with the framing effect (Tversky & Kahneman, 1981), and equalizes the impact of good and bad choice outcomes, thus modulating loss aversion (Tversky & Kahneman, 1992). Note that, in real settings, decisions are often sequenced together such that good or bad outcomes of given trials influence subsequent choices (Osborn & Jackson, 1988; Thaler & Johnson, 1990) and this can happen even when decision outcome is unpredictable or random as, for example, in the case of the Hot Hand Fallacy, in which random events with a positive outcome are wrongly interpreted as reflecting a winning streak (Ayton & Fischer, 2004).

Gao et al. (2015) tested whether risk taking would be modulated by language‐based feedback when participants repeatedly chose between playing and leaving (not playing) 50/50 gambles to win small monetary rewards. The choices were presented in numeric form, but the outcome was presented using adjectives with positive and negative valence in the participants’ first language (Chinese) or second language (English). We modeled the effects of presenting feedback, indicating good and bad outcome in either Chinese or English, upon participants’ subsequent decisions to play. In addition, we used ERPs to investigate a possible link between the neural correlates of language processing and the processing of decision outcomes themselves based on modulation of the feedback‐related negativity (FRN). The FRN is a frontally distributed negative deflection of ERPs, which typically peaks between 250 and 300 milliseconds after the onset of feedback stimulus and is sensitive to feedback valence, that is, how good or bad outcomes turn out to be (Gehring & Willoughby, 2002; Holroyd & Coles, 2002; Miltner, Braun, & Coles, 1997). Because emotional sensitivity has been shown to differ in the first and second language of bilinguals (Harris, Aycicegi, & Gleason, 2003), we hypothesized that feedback words in English would elicit an FRN smaller in amplitude than their Chinese equivalents, in turn affecting risk‐taking behavior. While positive feedback incited participants to play more on the next trial than negative feedback, we found a striking dissociation between languages, such that positive feedback in the native language (Chinese) incited participants to take 10% more gambles (i.e., more risk) on the next trial, as compared to all other conditions. This striking effect of language context was further supported by correlations between differences in FRN amplitude between language of feedback and differences in probabilities of playing. In other words, expressions that appear to convey the same message appear to have a profoundly different effect on decision making, depending on whether they are presented in the native or the second language of bilinguals, showing that interactions between language and decision making should not be taken lightly.

## Conclusion

There is now substantial neurophysiological evidence validating psycholinguistic data and theoretical linguistic accounts of linguistic relativity: Language(s), human perception, and aspects of cognition that may be construed as nonverbal interact in a rich and complex fashion. At the forefront of the current inquiry in this field is the question of the causal role of language and the depth of its pervasive influence on thinking. The studies reviewed above show that lexical and grammatical distinctions between languages affect elementary aspects of color and object perception, or motion conceptualization, and support the positive interpretation of results from behavioral experiments that have tested premises of the linguistic relativity hypothesis. Notwithstanding the debate among linguists regarding the existence of language universals (Evans & Levinson, 2009; von Fintel & Matthewson, 2008), a neurolinguistic approach can offer answers without requiring systematic comparison of all the many languages of the world. In addition to providing insights from within a particular language, the neurolinguistic approach allows us to characterize the nature of the relationship between formal aspects of language, perception, and concepts on the basis of unbiased physiological measurements rather than human performance in behavioral tasks prone to inciting metacognitive evaluation and thus susceptible to interpretative muddling.

Many questions remain unanswered. In fact, accepting the linguistic relativity hypothesis raises many new and fascinating questions: Is language and its particular forms not only sufficient, but also essential to the existence of certain perceptual distinctions? How reliable are such distinctions without language encoding? Are different types of learning—implicit or explicit—differently affected by language? How does language interact with other extralinguistic variables in shaping human cognition? How are the effects of differing linguistic codes reconciled in the multilingual mind? Does language proficiency directly or indirectly impact conceptual organization? How do languages and sociocultural conceptions interact in shaping human civilization? One can only hope that future research will progressively unveil such mechanisms and establish how strategic language use can better assist human learning, cognitive development, and decision making.
